# Comparative Effects of Exogenous Organic Amendments on Rhizosphere Microbial Communities and Soil Properties in Continuous Cropping Watermelon

**DOI:** 10.3390/microorganisms14040837

**Published:** 2026-04-08

**Authors:** Wen Pan, Li Gao, Yanjun Xu, Hongmei Guo, Ainiwar Abdulla, Alim Abdurim, Xiangyu Liu, Xingwang Gao, Haibo Wu

**Affiliations:** 1 Turpan Experimental Station, Academy of Agricultural Sciences of Xinjiang Uyghur Autonomous Region, Turpan 838000, China; 18199869974@163.com (W.P.); xyj9636@126.com (Y.X.); ghm@xaas.ac.cn (H.G.); 13289951650@163.com (A.A.); alim18909951191@163.com (A.A.); lxy791021@xaas.ac.cn (X.L.); 2 College of Life Sciences, Xinjiang University, Urumqi 830000, China; 17393068582@163.com (L.G.); gxw@xju.edu.cn (X.G.)

**Keywords:** watermelon production, organic fertilizer, exogenous amendments, continuous cropping, soil fertility, microbial diversity

## Abstract

Continuous cropping obstacles in watermelon are closely linked to rhizosphere microbial imbalance, posing a major threat to the sustainability of the industry in Xinjiang. Exogenous additives are widely used to regulate soil health, yet comprehensive comparisons of their mechanisms and effects remain limited. In this study, a field experiment was conducted under continuous watermelon cropping conditions in Xinjiang to evaluate the impact of eight treatments, including chemical fertilizer (NPK) alone and its combination with organic fertilizer (NPKM), glucose (NPKG), oxalic acid (NPKOA), amino acids (NPKGA), citric acid (NPKCA), and acetic acid (NPKAA), with unfertilized soil as the control (CK). Treatment effects were assessed through soil physicochemical analysis, fruit quality evaluation, and high-throughput sequencing (16S rRNA and ITS). Among all treatments, NPKM showed the greatest improvement in soil fertility, increasing soil organic matter by 13.91%, total nitrogen by 23.08%, and single fruit weight by 35.75% compared to CK. NPKGA also enhanced fruit weight (+33.06% vs. CK) and increased catalase activity, while oxalic acid exhibited the strongest activation of alkaline phosphatase. Microbiome analysis revealed that NPKM and NPKAA significantly reshaped both bacterial and fungal community structures. NPKM enriched beneficial taxa such as unclassified Chitinophagaceae and *Lophotrichus*, whereas NPKCA enriched the biocontrol bacterium *Pseudomonas chlororaphis*. Soil organic matter and total nitrogen were identified as key environmental drivers, showing significant positive correlations with core bacterial genera (*Dokdonella*) and negative correlations with the pathogenic fungus *Alternaria*. Collectively, this study elucidates the distinct mechanisms of various additives by linking treatment-specific microbial shifts to key soil factors and crop performance, providing a theoretical and technical framework for mitigating watermelon continuous cropping obstacles through rhizosphere environmental regulation.

## 1. Introduction

The watermelon (*Citrullus lanatus* (Thunb.) Matsum. & Nakai) is a major cash crop in China. In 2023, China accounted for 46.71% of the global watermelon cultivation area and 60.03% of global production. The Turpan region in Xinjiang is China’s primary area for early-maturing watermelon production. It covers 7280 hectares and yields 327,600 tons [1,2]. However, as the industry expands, the challenges posed by continuous cropping in intensive farming systems have become increasingly prominent, now representing a major obstacle to the sustainable development of the watermelon industry. The occurrence of continuous cropping obstacles is closely linked to imbalances in the rhizosphere microbiome. Research indicates that prolonged continuous cropping deteriorates soil physicochemical properties, significantly reduces rhizosphere microbial diversity, and disrupts microbial community structures involved in carbon-nitrogen cycling and hormone synthesis [3,4]. This is accompanied by the accumulation of autotoxins, which ultimately inhibit watermelon plant growth and adversely affect fruit yield and quality [3,4].

As an active interface for plant–soil–microbe interactions, the rhizosphere microbiome directly influences crop health and soil fertility through its microbial community structure and function [5]. Studies indicate that, under continuous cropping conditions, bacterial diversity in watermelon rhizosphere soil initially decreases and then increases, while fungal diversity continues to decline. At the genus level, the relative abundance of beneficial bacteria such as *Sphingomonas* and *Lysobacter* decreases, while that of pathogens such as *Fusarium* increases significantly with successive cropping cycles [6]. The alterations in the composition of the microbial community have been demonstrated to diminish the resilience of soil biota, but also to indirectly affect the physiological status of plants by disrupting the efficiency of nutrient cycling.

The rhizosphere microbiome can be defined as the integrated system comprising plant root systems and the microbial communities within the surrounding soil, along with their interactions. This phenomenon has been termed the ‘second genome’ of the plant. It has been demonstrated that the rhizosphere microbiome plays an instrumental role in providing essential nutrients to plants, including nitrogen, phosphorus, and potassium. In addition to this primary function, it has also been shown to enhance plant stress resistance and environmental adaptability by regulating the structure of the microbial community [7,8]. Research indicates that judicious intervention in the rhizosphere microbiome not only enhances soil nutrient availability but also optimizes microbial community structure, thereby boosting crop productivity. For instance, the application of nitrogen and phosphorus fertilizers to alfalfa (*Medicago sativa*) has been shown to significantly impact the physicochemical properties of the rhizosphere soil and its microbial community structure [9]. Inoculation with plant-growth-promoting rhizobacteria (PGPR) has been demonstrated to increase plant biomass and optimize microbial community composition [9]. The combined application of exogenous mycorrhizal fungi (e.g., *Boletus edulis*) with plant growth regulators such as naphthaleneacetic acid (NAA) and salicylic acid (SA) as rhizosphere microbial modulators has been demonstrated to significantly enhance poplar root vigor, increase soil acid phosphatase activity, and markedly elevate the content of soil available phosphorus (with increases ranging from 7.49% to 53.50%) [10]. Consequently, contemporary agroecological research has focused on exploring methods to enhance soil health and optimize watermelon yield and quality through the targeted regulation of the rhizosphere microbiome.

The utilization of exogenous organic additives is an effective strategy to regulate the rhizosphere microbiome. These additives include organic fertilizers, simple carbon sources, organic acids, and amino acids. It has been demonstrated that they indirectly promote crop growth and development. This promotion occurs through the modification of soil physicochemical properties and the modulation of the structure and function of microbial communities. For instance, organic fertilizers are rich in organic matter and nutrients, thereby providing abundant substrates to soil microorganisms. Consequently, these fertilizers increase the diversity of microbial communities in the rhizosphere [11,12]. Despite their seemingly elementary structural characteristics, simple carbon sources such as glucose have been shown to rapidly stimulate soil microbial activity and enhance the efficiency of biological nitrogen sequestration [13]. Organic acids exhibit more complex mechanisms of action. For instance, citric acid suppresses the growth of specific microorganisms by lowering the pH of the rhizosphere and facilitating the proliferation of acidophilic species [14]. Similarly, oxalic acid significantly increases the bioavailability of metals such as iron and aluminum due to its strong chelating capacity, thereby indirectly influencing microbial metabolic networks [15]. Furthermore, amino acids, which serve as high-quality nitrogen sources, have been shown to induce alterations in microbial carbon-nitrogen metabolism, thereby resulting in a functional reorganization of microbial communities [16].

Despite the considerable regulatory potential of these additives in the rhizosphere microbiome, current research on their application in modulating watermelon rhizosphere microbiology has identified several critical scientific questions that warrant further investigation. Firstly, the majority of studies have focused exclusively on the effects of single additives, and there is a lack of systematic comparative analyses involving different types of exogenous substances. Secondly, under actual field conditions, the mechanisms by which different additives drive the restructuring of microbial communities—through altering key rhizosphere factors such as pH, electrical conductivity, and nutrient availability—remain insufficiently understood. Thirdly, the underlying associations between shifts in microbial community and watermelon plant morphogenesis (e.g., main vine length and stem diameter) and fruit quality parameters (e.g., sugar and vitamin C content) remain inadequately understood.

In order to address the critical knowledge gaps identified, this study systematically investigates the effects of exogenous additives on the watermelon rhizosphere microbiome under continuous cropping systems in Xinjiang by employing eight distinct treatment regimens. By integrating multi-omics data, this research aims to elucidate three fundamental aspects of the relationship between exogenous additives and rhizosphere ecology: (1) the distinct regulatory patterns exerted by various additives on microbial activity; (2) the dynamic coupling mechanism between shifts in microbial biomass and soil nutrient release; and (3) the intrinsic associations among key microbial taxa, soil physicochemical properties, and crop performance.

Based on previous literature, we hypothesized that: (H1) organic fertilizer is more effective than simple organic compounds in promoting soil fertility; (H2) different additives select for distinct microbial communities, with organic fertilizer enriching beneficial bacteria while organic acids and amino acids predominantly affect fungi and enzymes; and (H3) shifts in key microbial taxa are directly associated with alterations in soil properties (particularly OM and TN), which are expected to correlate with enhanced crop performance.

## 2. Materials and Methods

### 2.1. Field Site and Experimental Design

Field trials were conducted from 2024 to 2025 at the Dongkan Experimental Station (42°54′22″ N, 89°19′17″ E) in Turpan City, Xinjiang Uygur Autonomous Region, China. The following properties were measured at the start of the experiment: pH, 8.50; water-soluble salt, 0.91 g/kg; alkali-hydrolysable nitrogen, 46.50 mg/kg; available phosphorus, 15.50 mg/kg; available potassium, 228.00 mg/kg; organic matter, 11.96 g/kg; total nitrogen, 0.58 g/kg.

The trial comprised eight treatment groups, as summarized in Table 1. The sheep manure applied in this study was sourced from a local farm and subjected to composting for approximately 3 months before use. The composting process involved regular turning at two-week intervals to ensure adequate conditions and facilitate uniform decomposition. The manure was evenly applied into furrows (excavated at a depth of approximately 15–20 cm) and then immediately incorporated into the soil by rotary tillage to ensure consistent mixing with the plow layer.

The experiment employed a randomized complete block design with three blocks. Each plot measured 8 m × 12 m (96 m^2^), with a 1-m-wide buffer zone between adjacent plots. Plants were spaced at 0.5 m intervals within rows, with a row spacing of 1.6 m. Within each block, the eight treatments were randomly assigned to plots, resulting in a total of 24 plots (8 treatments × 3 replicates). The watermelon cultivar used was ‘Sweetness 12K’ from the Daitianwang series.

Irrigation was scheduled based on crop growth stages: every four days during the vine extension period, regulated during flowering and fruit setting, and every two days during the fruit expansion stage. Irrigation was stopped 7–10 days before harvest. Plants were trained using the three-vine training system, retaining the main vine and two robust lateral vines. Fruit thinning was performed when young fruits reached the size of an egg, retaining one fruit per plant (preferably on the main vine) with 3–5 leaves remaining above the fruit node. All management practices were implemented uniformly across all plots.

### 2.2. Sample Collection

During the fruit ripening stage, three plants with similar growth conditions were randomly selected from each plot to collect leaf, stem, and fruit samples. Leaf and stem samples were thoroughly rinsed with distilled water, then dried at 105 °C for 30 min and subsequently oven-dried at 75 °C. For rhizosphere soil collection, entire watermelon plants were carefully excavated to preserve roots with their adhering soil. After removing the aboveground portion, the loosely attached soil was gently shaken off. Soil firmly adhering to the root surface was then collected using a sterile brush, passed through a 2-mm sieve [21], and defined as rhizosphere soil. Rhizosphere soil samples from the selected plants within each plot were composited, immediately placed on dry ice, and transported to the laboratory. Upon arrival, samples were divided: one portion was air-dried for the analysis of physicochemical properties and soil enzyme activities, and the remainder was stored at −80 °C for subsequent high-throughput sequencing.

In total, 24 composite rhizosphere soil samples (8 treatments × 3 replicates) were collected for soil physicochemical analysis, enzyme activity assays, and high-throughput sequencing analyses. For fruit quality analysis, 24 composite fruit samples (8 treatments × 3 replicates) were also obtained, with each composite sample comprising three fruits from the same plot.

### 2.3. Determination of Morphometric Parameters

We measured the main vine length, stem diameter, position of fruit-setting node, internode length, fresh stem weight, dry stem weight, fresh leaf weight, and dry leaf weight of the selected plant samples.

### 2.4. Fruit Quality Assessment

The following quality parameters of the selected fruit samples were determined. Individual fruit weight was recorded. The contents of individual sugar components (glucose, sucrose, and fructose) and vitamin C were analyzed using high-performance liquid chromatography (HPLC). Reducing sugar content was determined by the 3,5-dinitrosalicylic acid (DNS) colorimetric method. Total acidity was measured by acid-base indicator titration, and the soluble solids content (SSC) was assessed by refractometry. Fruit moisture and dry matter content were determined by the distillation method. Fruit protein content was quantified by the Kjeldahl method. Potassium and phosphorus contents were determined by flame photometry and the ammonium vanadomolybdate spectrophotometric method, respectively [22,23,24,25,26].

### 2.5. Determination of Physicochemical Properties of Rhizosphere Soil

Soil physicochemical properties were determined according to standard protocols. Soil pH was measured by potentiometric titration [27]. Water-soluble salts (WSS) were quantified gravimetrically [28]. Alkali-hydrolysable nitrogen (AN) was determined by the diffusion absorption technique [29]. Available phosphorus (AP) was extracted with sodium bicarbonate and measured by the molybdenum antimony anti-colorimetric method [30]. Available potassium (AK) was extracted with ammonium acetate and quantified by flame photometry [31]. Organic matter (OM) was determined using the potassium dichromate oxidation–titration method [32]. Total nitrogen (TN) was analyzed using an automatic nitrogen analyzer [32]. Microbial biomass carbon (MBC) and nitrogen (MBN) were measured by the chloroform fumigation Kjeldahl method and chloroform fumigation–Kjeldahl method, respectively [32].

### 2.6. Soil Enzyme Activity Assay in the Rhizosphere

The activities of sucrase (S-SC), urease (S-UE), catalase (S-CAT), and alkaline phosphatase (S-AKP/ALP) in rhizosphere soil were determined using microassays [33].

### 2.7. Soil DNA Extraction and PCR Amplification

Total microbial DNA was extracted from soil samples using the E.Z.N.A.^®^ Soil DNA Kit (Omega Bio-tek, Norcross, GA, USA) according to the manufacturer’s protocol. DNA concentration and purity were assessed by 1% agarose gel electrophoresis and a NanoDrop™ 2000 spectrophotometer (Thermo Fisher Scientific, Wilmington, DE, USA). To monitor potential contamination during DNA extraction and PCR amplification, negative controls were systematically incorporated. Specifically, extraction blanks (samples processed without soil) were included alongside the soil samples, and PCR negative controls (nuclease-free water instead of template DNA) were included in each amplification run. No detectable amplification products were observed in any negative controls, confirming the absence of contamination. Bacterial and fungal libraries were constructed independently. The V3–V4 hypervariable region of the bacterial 16S rRNA gene was amplified using the universal primers 338F (5′-ACTCCTACGGGGCAGG-3′) and 806R (5′-GGACTACHVGGGTWTCTAAT-3′) [34]. The fungal ITS1 region was amplified using the universal fungal primers ITS1F (5′-CTTGGTCATTTAGAGGAAGTAA-3′) and ITS2R (5′-GCTGCGTTCTTCATCGATGC-3′) [35]. PCR amplification was performed under the following conditions: initial denaturation at 95 °C for 3 min; 27 cycles of denaturation at 95 °C for 30 s, annealing at 60 °C for 30 s, and extension at 72 °C for 30 s; and a final extension at 72 °C for 10 min. The PCR products were then quantified using the QuantiFluor™-ST Blue Fluorescent Quantification System (Promega, Madison, WI, USA) and purified with the AxyPrep DNA Gel Purification Kit (Axygen Biosciences, Union City, CA, USA). Purified amplicons were pooled in equimolar amounts and paired-end sequenced on an Illumina NextSeq 2000 platform (Illumina, San Diego, CA, USA) according to the standard protocols by Majorbio Bio-Pharm Technology Co., Ltd. (Shanghai, China). After quality filtering, an average of 50,000 high-quality reads per sample was obtained.

### 2.8. Sequencing Data Analysis

Raw paired-end FASTQ files were processed by Shanghai Majorbio Bio-Pharm Technology Co., Ltd. (Shanghai, China) for sample demultiplexing and preliminary quality control. The reads were quality-filtered using fastp (version 0.19.6) [36] and merged by FLASH (version 1.2.7) [37]. After primer removal and elimination of low-quality reads, high-quality sequences were dereplicated, screened for chimeras, and clustered into operational taxonomic units (OTUs) using the UPARSE algorithm (USEARCH v11; http://drive5.com/uparse; accessed on 14 June 2025) at a 97% sequence similarity threshold, which incorporates built-in error reduction to minimize spurious clusters [38,39]. The most abundant sequence in each OTU was retained as the representative sequence for subsequent analyses. To remove non-target reads, sequences annotated as chloroplasts or mitochondria were excluded from the OTU table following taxonomic assignment [39]. To normalize sequencing depth across samples, rarefaction was performed by randomly subsampling 40,000 sequences per sample, which was slightly below the minimum read count (42,000 reads) after quality filtering, ensuring >99% coverage for all samples. Taxonomic assignment of OTU representative sequences was performed using the RDP naïve Bayesian classifier (version 11.5) with a confidence threshold of 0.7. Bacterial 16S rRNA gene sequences were classified against the SILVA v138 16S rRNA reference database, whereas fungal ITS sequences were annotated using the UNITE database (version 9.0) [40,41].

### 2.9. Data Statistics and Analysis

Statistical analyses were performed as follows. One-way analysis of variance (ANOVA) was conducted using SPSS 24.0 (IBM Corp., Armonk, NY, USA) to test for significant differences among treatments (*p* < 0.05), followed by Tukey’s honestly significant difference (HSD) post hoc test for multiple comparisons where applicable. All high-throughput sequencing data were processed and analyzed on the MajorBio Cloud Platform (https://cloud.majorbio.com; accessed on 14 September 2025). Alpha diversity indices (Shannon diversity) were calculated using mothur (version 1.48.0; http://www.mothur.org; accessed on 14 September 2025). The Simpson index is reported in its original form (D = Σp_i_^2^), where a higher value reflects lower diversity (i.e., reduced evenness). Differences in alpha diversity between groups were evaluated using the Wilcoxon rank-sum test. Beta diversity was assessed by principal coordinate analysis (PCoA) based on Bray–Curtis dissimilarity matrices. Permutational multivariate analysis of variance (PERMANOVA) with 999 permutations was applied to test for significant differences in community structure between groups. Linear discriminant analysis effect size (LEfSe; http://huttenhower.sph.harvard.edu/lefse/; accessed on 14 September 2025) was employed to identify bacterial and fungal taxa exhibiting significantly different relative abundances among groups from the phylum to genus levels (linear discriminant analysis [LDA] (score > 3, *p* < 0.05) [42]. Distance-based redundancy analysis (db-RDA) was performed to evaluate the relationships between soil physicochemical properties and microbial community composition. Furthermore, linear regression analysis was used to assess the effects of key soil environmental variables identified by db-RDA on microbial alpha diversity indices [43,44,45]. The raw sequencing data have been submitted to the NCBI Sequence Read Archive (SRA) (accession number: PRJNA945192).

## 3. Results

### 3.1. Effects of Different Exogenous Additives on Physiological Parameters of Watermelon

**Figure 1:**
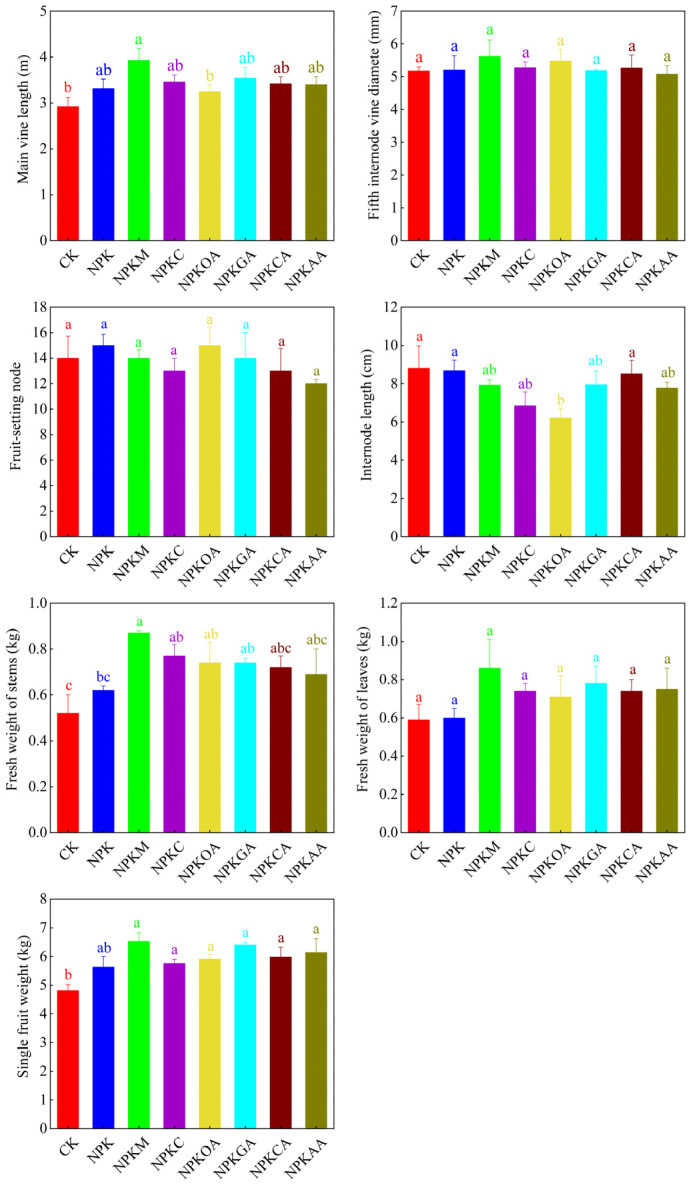
Morphometric parameters of watermelon under different treatments. Values with different letters are significantly different at *p* < 0.05.

As shown in Figure 1, all fertilization treatments significantly increased watermelon fruit weight (*p* < 0.05) compared to the unfertilized CK. Of these treatments, NPKM had the most pronounced effect, with a mean fruit weight of 6.53 kg, which was significantly greater than those observed in the CK and NPK treatments (increases of 35.75% and 15.99%, respectively). The amino acid (NPKGA) treatment also exhibited favorable results, with a mean fruit weight of 6.40 kg (increases of 33.06% and 13.68% compared to CK and NPK, respectively). Regarding plant growth, fertilization treatments similarly exhibited significant promotional effects (Figure 1). The main vine length and fresh stem weight of the NPKM treatment were the highest of all the treatments and significantly exceeded those of the control. The main vine length and fresh stem weight of the remaining fertilization treatments (e.g., NPKC, NPKGA, and NPKAA) were also significantly higher than the control, indicating that the addition of external substances effectively promoted plant growth. While fertilization treatments significantly influenced vegetative growth and yield, their impact on certain morphological traits was minimal. No significant differences (*p* > 0.05) were observed among treatments for fifth internode vine diameter, fruiting node position, or fresh leaf weight.

### 3.2. Effects of Different Exogenous Additives on Watermelon Fruit Quality

As shown in Table 2, different fertilization treatments had no significant effect (*p* > 0.05) on the fundamental quality indicators of watermelon fruit, such as moisture content, dry matter, soluble solids, vitamin C content, protein content, and phosphorus content. However, significant differences in sugar composition emerged across treatments. Specifically, NPKM had the most pronounced effect on sucrose accumulation in fruit, while the oxalic acid (NPKOA) treatment increased, reducing sugar content. Conversely, the NPK treatment, which utilized only chemical fertilizer, had lower fructose and glucose contents than most other treatments. In summary, fertilization treatments, particularly those involving the combined application of organic and inorganic fertilizers such as NPKM, can effectively optimize sugar metabolism in watermelon fruit, significantly increasing sucrose content while exerting minimal influence on other fruit characteristics.

### 3.3. Soil Physicochemical Properties in Response to Different Exogenous Additives

**Figure 2:**
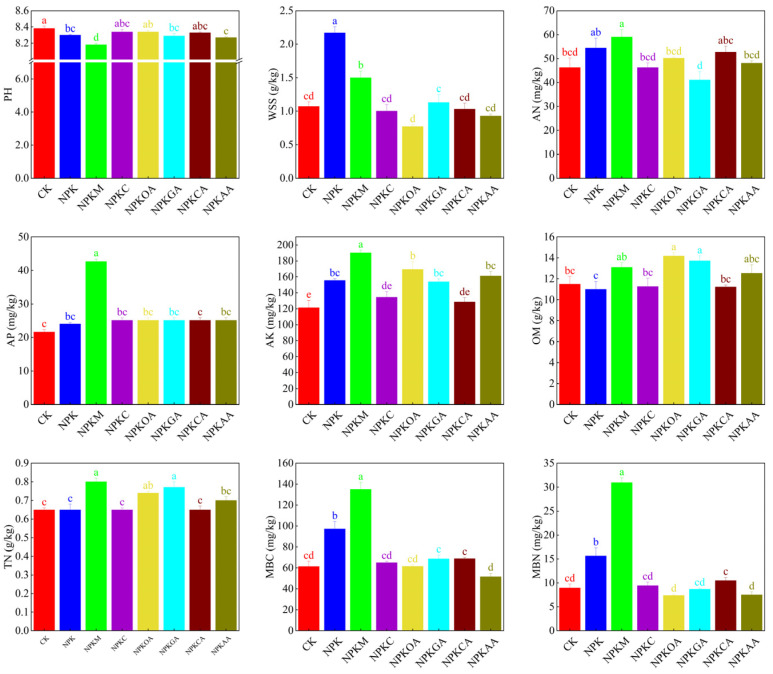
Soil Physicochemical Properties Analysis. pH: hydrogen ion concentration; WSS: Water-soluble salts; AN: Alkali-hydrolysable nitrogen; AP: Available phosphorus; AK: Available potassium; OM: Organic matter; TN: Total nitrogen; MBC: Microbial biomass carbon; MBN: Microbial biomass nitrogen. Values with different letters are significantly different at *p* < 0.05.

Different exogenous additives had varying effects on soil physicochemical properties (e.g., pH, water-soluble salts, and nutrient content) and on microbial activity (Figure 2). Compared with NPK and CK, the organic fertilizer treatment NPKM exhibited the most comprehensive effects. This treatment not only significantly reduced soil pH, alleviating soil alkalinity, but also increased soil organic matter and total nitrogen contents to 13.10 g/kg and 0.80 g/kg, respectively. Concurrently, it substantially increased soil microbial biomass carbon and microbial biomass nitrogen, indicating that this treatment effectively enhances soil biological activity. In contrast, NPK increased available nitrogen and available potassium levels, but it also led to water-soluble salt accumulation reaching 2.17 g/kg, thereby posing a risk of salinization. The NPKOA and NPKGA treatments demonstrated outstanding performance in enhancing organic matter, increasing it to 14.17 and 13.73 g/kg, respectively, while the NPKAA treatment showed significant efficacy in lowering soil pH.

### 3.4. Enzyme Activity in Watermelon Rhizosphere Soil in Response to Different Exogenous Additives

**Figure 3:**
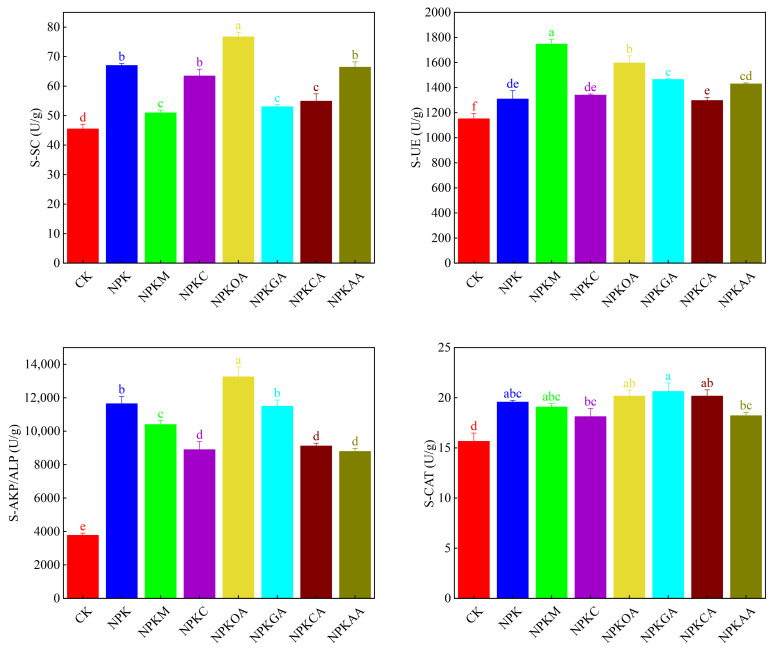
Soil Enzyme Activity Analysis. S-SC: Soil sucrase; S-UE: Soil urease; S-AKP/ALP: Soil alkaline phosphatase; S-CAT: Soil catalase. Values with different letters are significantly different at *p* < 0.05.

Analysis of soil enzyme activity revealed that different fertilization treatments exerted significant effects on soil biochemical processes (Figure 3). The NPKOA treatment demonstrated the most pronounced enhancement in soil sucrase and alkaline phosphatase activity, with values significantly exceeding those of the other treatments. The NPKM treatment markedly increased soil urease activity, rising by 51.8% compared to the CK, indicating its strongest capacity to promote nitrogen transformation. The NPKGA treatment exhibited the highest catalase activity, alongside relatively elevated alkaline phosphatase activity. NPKC and NPKAA treatments also exhibited promotive effects on sucrase and alkaline phosphatase, although the overall increase was less pronounced than that observed with NPKOA and NPKGA treatments.

### 3.5. Changes in the Microbial Community Structure of Watermelon Rhizosphere Soil

#### 3.5.1. Microbial Cluster Analysis

**Figure 4:**
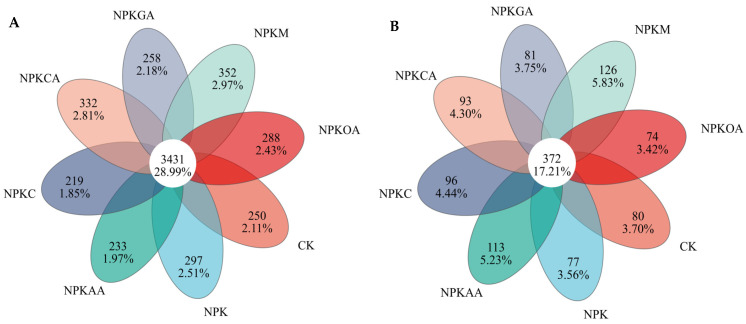
Venn diagrams illustrating unique and shared OTUs among treatments. (**A**) Bacterial community; (**B**) Fungal community. The Venn diagrams were generated using the Majorbio Cloud Platform (https://cloud.majorbio.com; accessed on 14 September 2025).

Venn diagrams were employed to analyze the bacterial and fungal community structures, respectively. The bacterial community structure is depicted in Figure 4A, where 3481 operational taxonomic units (OTUs) (28.99%) were common across all treatments, indicating that a substantial proportion of stable microbial communities persisted across different treatments. However, each treatment harbored a distinct number of unique OTUs. Among these, the NPKM treatment had the highest number of unique OTUs (352, accounting for 2.97%), followed by NPKCA (332, accounting for 2.81%). This indicates that the NPKM and NPKCA treatments exerted the most pronounced effects on soil microbial community structure, shaping distinct bacterial assemblages. Fungal community structure is depicted in Figure 4B, where 372 OTUs (17.21%) were shared across all treatments. Among these, the NPKM treatment harbored the highest number of unique OTUs (126, 5.83%), followed by NPKAA (113), NPKC (96), NPKCA (93), NPKGA (81), CK (80), NPK (77), and NPKOA (74). Results suggest that different exogenous additives can influence soil microbial composition.

#### 3.5.2. Effects of Different Exogenous Additives on Soil Microbial Diversity

**Figure 5:**
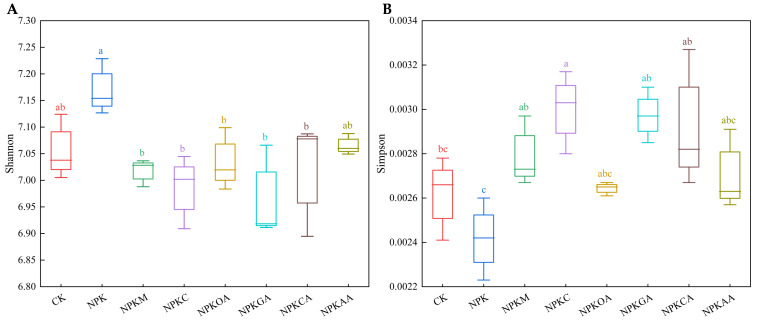
Alpha diversity of bacterial communities under different treatments. (**A**) Shannon index; (**B**) Simpson index. Different lowercase letters indicate significant differences (*p* < 0.05).

The Shannon and Simpson indices were used to evaluate community diversity. As depicted in Figure 5, the Shannon and Simpson indices varied among treatments. Except for the NPKOA and NPKAA treatment groups, the Shannon index decreased, and the Simpson index increased in all other treatment groups. Nevertheless, no significant differences were observed among the treatment groups. These results indicate that different exogenous additives did not have a significant effect on bacterial community richness in rhizosphere soil but did influence bacterial community diversity.

**Figure 6:**
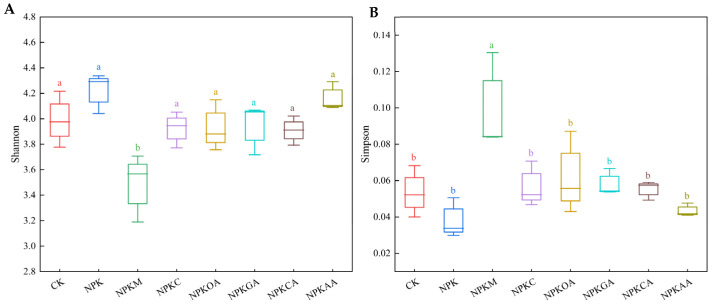
Alpha diversity of fungal communities under different treatments. (**A**) Shannon index; (**B**) Simpson index. Different lowercase letters indicate significant differences (*p* < 0.05).

Fungal community diversity indices (Figure 6) revealed that the NPKM treatment resulted in a significant increase in the Simpson index and a concurrent significant decrease in the Shannon index (indicators of community diversity) compared to the control (CK) and NPK groups, suggesting that this treatment markedly altered fungal community diversity by enhancing the dominance of key species. No significant changes in diversity indices were detected among the other treatment groups. In summary, exogenous additives had no significant effect on fungal community richness, but the addition of organic fertilizer significantly altered fungal community diversity.

**Figure 7:**
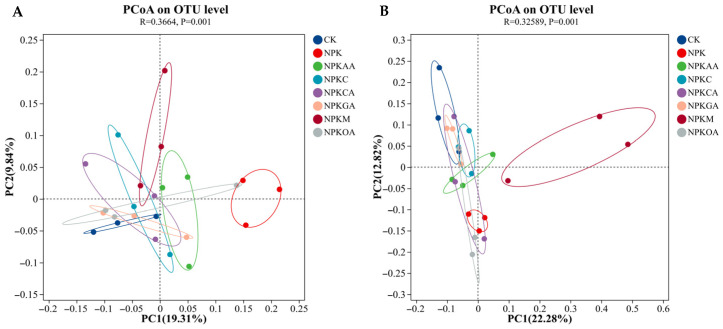
PCoA of microbial communities based on using Bray–Curtis dissimilarity. (**A**) Bacterial community; (**B**) fungal community.

Based on OTU β-diversity comparisons, Principal coordinate analysis (PCoA) ordination, and analysis of similarities (ANOSIM) were conducted to assess differences among treatments. The bacterial PCoA is depicted in Figure 7A, where the NPKM, NPKOA, and NPKAA treatment clusters are distinctly separated from CK and NPK, indicating that organic fertilizer, oxalic acid, and acetic acid exert significant effects on bacterial community structure. The fungal PCoA is depicted in Figure 7B, where the NPKM and NPKAA treatment clusters are markedly separated from CK and NPK, indicating that organic fertilizer and acetic acid significantly influence fungal community structure.

#### 3.5.3. Microbial Species Composition Analysis

**Figure 8:**
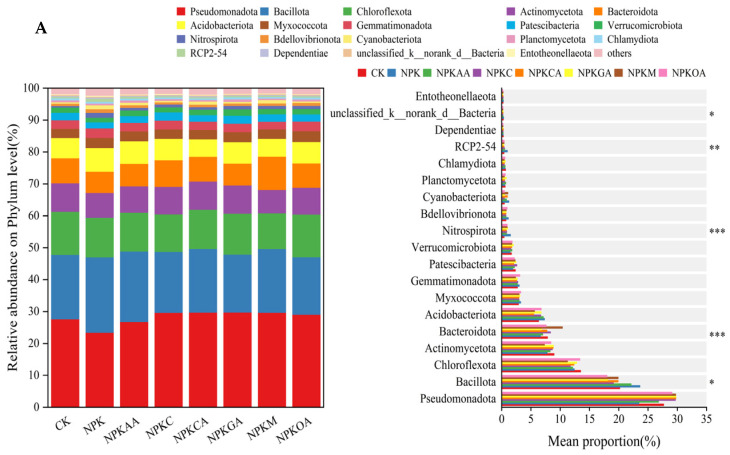

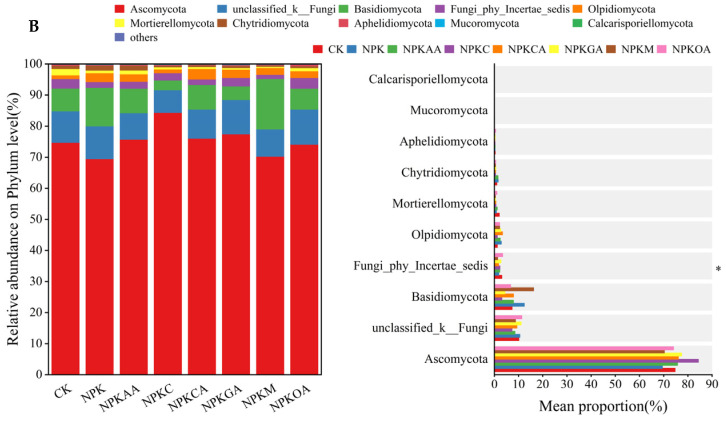
Phylum-level differences in community abundance for (**A**): soil bacterial community and (**B**): soil fungal community. * indicates significance at *p* < 0.05, ** at *p* < 0.01, and *** at *p* < 0.001. Taxa labeled with prefixes such as ‘unclassified’ represent sequences that could not be assigned to a named taxon and are shown at the highest resolved taxonomic level.

Analysis of rhizosphere bacterial communities at the phylum level across different treatment groups (Figure 8A) revealed that the dominant bacterial phyla were Pseudomonadota, Bacillota, Chloroflexota, and Actinomycetota. These phyla were predominant in all treatments and constituted the main components of the rhizosphere bacterial communities. Compared to the CK and NPK treatments, the addition of different exogenous additives resulted in distinct differences in the community structure. Specifically, the NPKGA and NPKOA treatments significantly reduced the relative abundance of Bacillota; in contrast, the NPKM treatment markedly increased the relative abundance of Bacteroidota; moreover, the relative abundances of other bacterial taxa did not differ significantly among treatments.

Analysis of fungal communities at the phylum level in rhizosphere soils across different treatments is presented in Figure 8B. Ascomycota emerged as the dominant fungal phylum in all treatments, with relative abundances exceeding 50% in each case, particularly reaching the highest proportion in the NPKC treatment. Compared to the CK, NPK treatments reduced the relative abundance of unclassified k-Fungi while increasing that of Basidiomycota. Concurrently, different exogenous additive treatments exerted differential effects on fungal communities: the NPKM treatment significantly promoted Basidiomycota growth, whereas the NPKC and NPKG treatments reduced their relative abundance.

**Figure 9:**
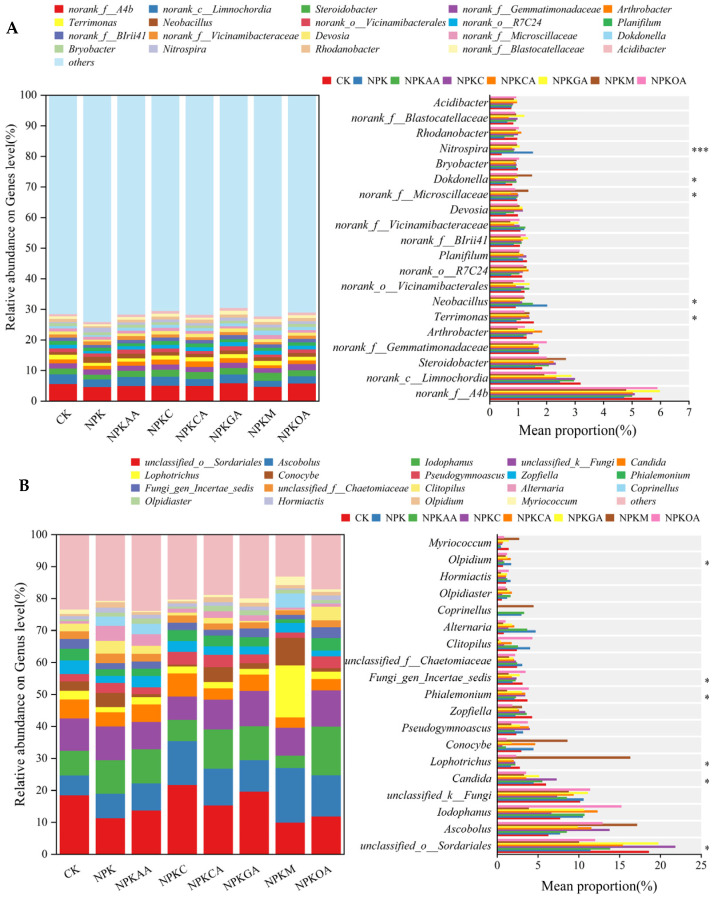
Taxonomic composition of bacterial and fungal communities at the genus level and above. (**A**) soil bacterial community and (**B**) soil fungal community. * indicates significance at *p* < 0.05, and *** at *p* < 0.001. Taxa labeled with prefixes such as ‘unclassified’ or ‘norank’ represent sequences that could not be assigned to a named taxon and are shown at the highest resolved taxonomic level.

The taxonomic composition of rhizosphere bacterial and fungal communities at the genus level and above is presented in Figure 9. While exogenous additives exerted no significant influence on bacterial community structure, they exerted a marked effect on fungal community composition. For bacterial communities (Figure 9A), several classified genera and unclassified lineages were observed. Among the unclassified lineages, norank_f__A4b (representing a taxon classified only to the family level) emerged as the most dominant across all treatments, maintaining a consistently high relative abundance. Other abundant taxa included norank_c__Limnochordia (unclassified at the class level), the classified genus *Steroidobacter*, and norank_f__Gemmatimonadaceae (unclassified at the genus level). Among the classified genera, no significant differences in relative abundances were detected across treatments. For fungal communities (Figure 9B), unclassified_o__Sordariales (representing sequences classified only to the order level) was the most dominant taxon across all treatments, maintaining a consistently high relative abundance in each treatment. Compared to the CK and NPK treatments, the other fertilization treatments exhibited distinct differences in community structure. For instance, the relative abundance of *Ascobolus* was significantly increased across all treatments, while those of *Iodophanus* and *Candida* significantly decreased in the NPKM treatment. Notably, the relative abundance of *Lophotrichus* in the NPKM treatment increased significantly compared to all other treatment groups.

#### 3.5.4. Microbial LEfSe Analysis

**Figure 10:**
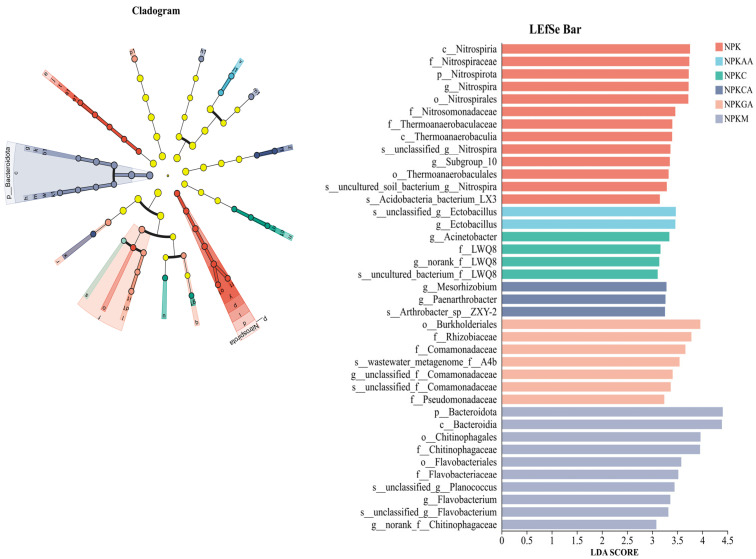
LEfSe showing differentially abundant bacterial taxa among treatments. The cladogram illustrates taxonomic hierarchies. Colors in both panels indicate different treatments. Taxa labeled with prefixes such as ‘unclassified’ or ‘norank’ represent sequences that could not be assigned to a named taxon and are shown at the highest resolved taxonomic level.

**Figure 11:**
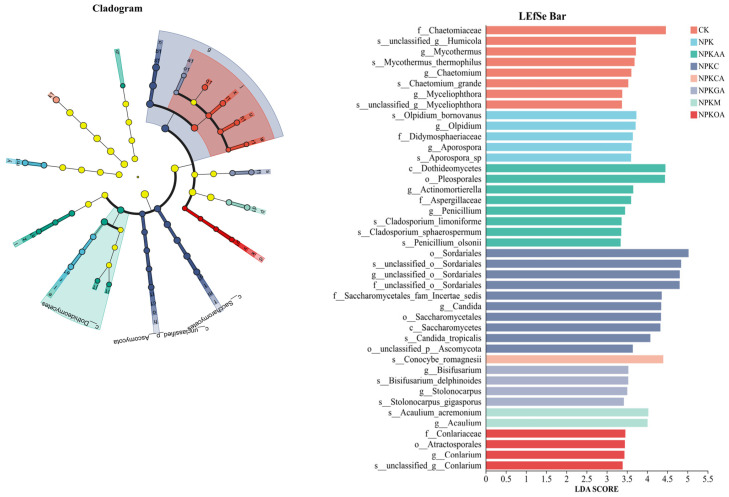
Changes in rhizosphere fungal communities of watermelon under different exogenous additive treatments. Colors in both panels indicate different treatments. Taxa labeled with prefixes such as ‘unclassified’ or ‘norank’ represent sequences that could not be assigned to a named taxon and are shown at the highest resolved taxonomic level.

Linear discriminant analysis (LEfSe) was employed to identify bacterial and fungal species that exhibited significant differences among treatment groups. Based on an LDA score > 3 and *p* < 0.05 for linear discrimination, the results indicated that distinct fertilisation treatments significantly shaped unique microbial communities, enriching microbial groups with varying phylogenetic classifications and ecological functions (Figure 10 and Figure 11). A total of 41 bacterial and 47 fungal taxonomic units were identified as significantly differential biomarkers among groups. Among these, the NPK treatment group was enriched in 10 significant bacterial biomarkers, including Nitrospirota, Nitrosomonadaceae, and Thermoanaerobaculia, as well as five significant fungal biomarkers, including Didymosphaeriaceae, *Olpidium*, and *Aporospora*. The NPKCA treatment group was enriched in eight significant bacterial biomarkers, including *Acinetobacter*, Pseudomonadaceae, and *Pseudomonas chlororaphis*, as well as one significant fungal biomarker *Conocybe romagnesii*; The NPKGA treatment group was enriched in seven significant bacterial biomarkers, including the phylum Bacteroidetes, and four significant fungal biomarkers, including *Stolonocarpus* and *Bisifusarium*; The NPKAA treatment group was enriched in two significant bacterial biomarkers, including the genus *Ectobacillus* and its related taxonomic units, as well as eight significant fungal biomarkers such as Dothideomycetes, Pleosporales, and Aspergillaceae; The NPKC treatment group was enriched in five significant bacterial biomarkers, including Subgroup_10, and ten significant fungal biomarkers such as Saccharomycetales, Candida, and Sordariales; The NPKM treatment group was enriched in 10 significant bacterial biomarkers, including an unclassified genus under the family Chitinophagaceae (Norank_f_Chitinophagaceae) and an unclassified genus under the family Comamonadaceae (g_unclassified_f_Comamonadaceae), as well as 2 significant fungal biomarkers, *Acaulium* and *Acremonium.* No significantly indicative bacterial taxonomic units were identified in CK or NPKOA, whereas eight (Didymosphaeriaceae, *Olpidium*, Aporospora, etc.) and four (Atractosporales, *Conlarium*, etc.) significant fungal biomarkers were detected in the fungal taxonomic units, respectively.

#### 3.5.5. Correlation Analysis Between Microorganisms and Soil Physicochemical Properties

**Figure 12:**
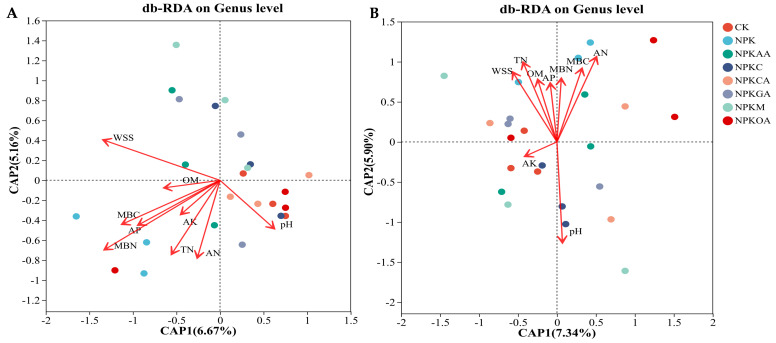
db-RDA showing the relationship between soil physicochemical properties and microbial community composition. Red arrows represent soil factors; arrow length indicates the strength of influence. (**A**) Bacterial community; (**B**) Fungal community.

The results of distance-based redundancy analysis (db-RDA) based on the genus-level microbial community structure are presented in Figure 12. CAP1 and CAP2 represent the principal coordinate axes explaining variation in community structure. In Figure 12A, CAP1 and CAP2 account for 6.67% and 5.16% of the variation in bacterial community structure, respectively. Soil bacterial communities are significantly correlated with water-soluble salts (WSS), microbial nitrogen content (MBN), and microbial carbon content (MBC). In Figure 12B, CAP1 and CAP2 explain 7.34% and 5.90% of fungal community structural variation, respectively. Soil fungal communities are significantly correlated with pH and available nitrogen (AN). This indicates that soil physicochemical properties are key factors influencing bacterial and fungal community structure.

**Figure 13:**
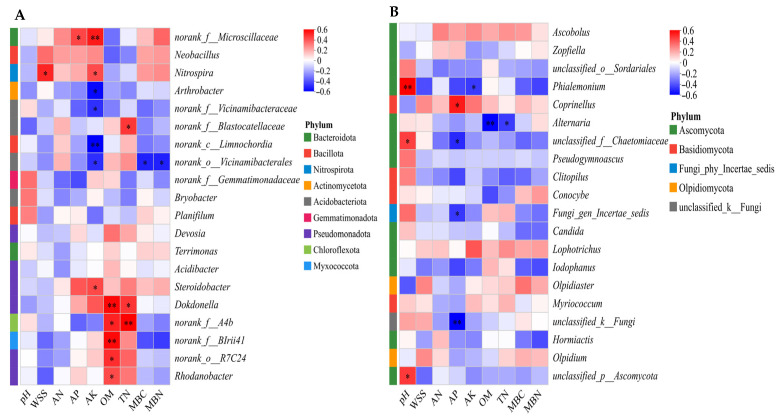
Heatmap showing Spearman correlations between soil physicochemical properties and microbial taxa. Color intensity represents correlation coefficients. (* *p* < 0.05; ** *p* < 0.01). (**A**): Bacterial community; (**B**): fungal community. The heatmap was generated using the Majorbio Cloud Platform (https://cloud.majorbio.com; accessed on 14 September 2025). Taxa labeled with prefixes such as ‘unclassified’ or ‘norank’ represent sequences that could not be assigned to a named taxon and are shown at the highest resolved taxonomic level.

Spearman’s rank correlation analysis was employed to examine the associations between soil physicochemical properties and variations in the abundance of core bacterial genus (as depicted in Figure 13A). Organic matter (OM) and total nitrogen (TN) jointly promoted the enrichment of Dokdonella and norank_f__A4b; available potassium (AK) exhibited broad effects: it showed significant positive correlations with water-soluble salts (WSS) and Nitrospira, as well as with available phosphorus (AP) and norank_f__Microscillaceae. Concurrently, it exerted significant negative effects on the relative abundance of norank_o__Vicinamibacterales, as well as on microbial biomass carbon (MBC) and microbial biomass nitrogen (MBN). Organic matter (OM) and available potassium (AK) are key soil factors influencing changes in core bacterial genera.

The association between soil physicochemical properties and the abundance of core fungal genera is detailed in Figure 13B. This correlation analysis revealed that pH had a significant positive effect on multiple Ascomycota-related fungi (e.g., unclassified_p_Ascomycota, unclassified_f_Chaetomiaceae) and *Phialemonium*; available phosphorus (AP) significantly promoted the growth of *Coprinellus* while inhibiting taxa such as unclassified_f_Chaetomiaceae; conversely, the relative abundance of *Alternaria* decreased significantly with increasing organic matter (OM) and total nitrogen (TN).

## 4. Discussion

### 4.1. Effects of Different Exogenous Additives on the Physicochemical Properties of Watermelon Rhizosphere Soil

The present study systematically evaluated the ameliorative effects of various exogenous additives on the physicochemical properties of root-zone soil in continuous watermelon cropping. Our results demonstrate that organic fertilizers exerted the most pronounced effects on overall soil fertility, with substantial increases in soil organic matter, total nitrogen content, and microbial carbon and nitrogen content. This finding confirms our first hypothesis (H1) that organic fertilizer is more effective than simple organic compounds in enhancing soil fertility. This finding is consistent with those of Song et al. [46], who observed that organic fertilizers create an environment conducive to microbial proliferation by supplying abundant organic substrates and nutrients, thereby enhancing soil nutrient cycling and energy flow. It is noteworthy that the NPKM treatment significantly reduced soil pH, which is particularly important in the context of ameliorating the widespread alkaline soils of Xinjiang. Such soils frequently immobilize substantial amounts of nutrients, thereby diminishing their bioavailability [47]. The utilization of organic fertilizers may contribute to mitigating soil alkalization, possibly due to the release of organic acids and the stimulation of microbial metabolism [48,49,50].

Oxalic acid and amino acids have been shown to increase soil organic matter, likely due to their function as readily degradable carbon and nitrogen sources that stimulate microbial metabolism and thereby promote organic matter accumulation [51,52]. Liu Yonghong et al. [53] provided further confirmation that the application of small-molecule organic acids to soil can enhance soil fertility through dual mechanisms: chelation and the provision of microbial substrates. However, the observation that oxalic acid outperformed the other organic acids tested provides novel insights for the development of new soil conditioners.

Conversely, conventional fertilization, while increasing the availability of nutrients, resulted in water-soluble salt accumulation reaching 2.17 g/kg, thereby posing a significant risk of salinization. This finding is consistent with the conclusions of Wen et al.’s [35] research, which indicate that the long-term application of chemical fertilizers can lead to secondary soil salinization. This emphasizes the significance of employing a combination of organic and inorganic fertilization methods to maintain soil health.

### 4.2. Effects of Different Exogenous Additives on Physiological Parameters and Quality of Watermelon

The enhancement effects of exogenous additives on watermelon growth and fruit quality are primarily reflected in biomass accumulation and fruit quality indicators. The NPKM and NPKGA treatments demonstrated optimal performance in terms of single fruit weight and plant biomass, likely attributable to their improvement of the rhizosphere microenvironment, thereby enhancing nutrient uptake efficiency and photosynthetic capacity [54,55]. From a physiological mechanism perspective, the utilization of organic fertilizers in conjunction with amino acids has the potential to stimulate plant growth through a variety of physiological pathways. These include the provision of a sustained nutrient supply via slow-release nutrients [56], the stimulation of root development to expand the absorption surface area [57], and the regulation of plant hormone balance to promote cell division and expansion [58].

Extensive research has confirmed that the combined application of organic and chemical fertilizers constitutes an effective strategy for enhancing fruit and vegetable quality. This approach has been demonstrated to enhance soil structure, promote root development and nutrient uptake, and thereby significantly increase fruit sugar content and potassium nutrition levels [59,60]. The present findings indicate that organic–inorganic combination treatments, such as NPKM and NPKOA, significantly optimize sugar composition and increase potassium content in watermelons. This finding is consistent with the previously reported positive effects observed in crops such as tomatoes and citrus [61,62].

However, the regulatory effects of organic fertilizers on fruit quality also exhibit certain crop and environmental dependencies. A substantial corpus of research suggests that organic fertilizers have the capacity to enhance the vitamin C content of crops such as tomatoes and apples [63,64,65]. However, the present study observed no significant effects across treatments on nutritional indicators, including vitamin C and protein content. This finding indicates that fertilization effects may be influenced by the interaction of multiple factors, including crop species, soil fertility, climatic conditions, and the source and application rate of organic fertilizers [66]. Partially confirming H3, the improved crop performance under organic fertilizer treatment was associated with shifts in microbial communities and enhanced soil properties, demonstrating the intrinsic linkages among additives, soil factors, microbial taxa, and crop phenotypes.

### 4.3. Effects of Different Exogenous Additives on Enzyme Activity in Watermelon Rhizosphere Soil

Compared with other exogenous additives in this study, oxalic acid-treated sucrose exhibited relatively higher alkaline phosphatase activity. This finding is consistent with the report by Fei et al. [67], who observed that oxalic acid effectively activates enzyme systems associated with soil phosphorus cycling. Concurrently, the study revealed that organic acids exerted a more significant enhancing effect on enzyme activity, particularly with oxalic acid exhibiting a far greater than anticipated activation of phosphatase. This phenomenon may be associated with the significant phosphorus fixation observed in the soils of Xinjiang, where the presence of oxalic acid has been shown to chelate calcium ions, thereby facilitating the release of immobilized phosphorus and the consequent activation of phosphatase activity [68]. The potent activation of phosphatase by oxalic acid demonstrates its substantial potential as an efficient phosphorus activator in severely phosphorus-fixed alkaline soils [69], thereby providing a theoretical basis for developing novel soil conditioners.

The application of organic fertilizers resulted in a substantial enhancement of urease activity, thereby signifying their remarkable efficacy in facilitating nitrogen transformation processes [70]. This phenomenon is closely associated with the ample nitrogen supply provided by organic fertilizers and their capacity to stimulate microbial activity [71]. It is noteworthy that amino acids exerted the most favorable effect on catalase activity, suggesting that amino acid supplementation may enhance soil redox capacity, thereby aiding free radical scavenging and maintaining cell membrane stability [72]. This study further revealed that organic acid additives (e.g., oxalic and citric acids) generally promoted enzyme activity more effectively than sugars (e.g., glucose), indicating that organic acids may confer greater advantages for phosphorus activation in alkaline soils. These changes in enzyme activity reflect enhanced soil nutrient transformation capacity and increased soil stress resistance.

### 4.4. Effects of Different Exogenous Additives on the Structure and Function of Microbial Communities in Watermelon Rhizosphere Soil

The analysis of microbial communities suggests that exogenous additives, particularly organic fertilizers and organic acids, significantly influence the structure and function of watermelon rhizosphere microbial communities. Venn diagram analysis revealed that each treatment generated varying numbers of unique OTUs, with organic fertilizer yielding the highest number of unique bacterial OTUs (352). Furthermore, PCoA demonstrated that this treatment was distinctly separated from the control group, indicating its most pronounced effect on microbial community structure. This finding is consistent with the conclusions of Shu et al. [73], which demonstrated that, across diverse agricultural systems worldwide, organic amendments significantly enhance soil microbial diversity. Our results extend this general principle by confirming its applicability specifically within the context of continuous watermelon cropping in the alkaline soils of Xinjiang. Concurrently, this study observed a markedly higher microbial response to organic acids than to sugars. The greatest separation from the control in PCoA was exhibited by oxalic acid and acetic acid treatments, indicating that organic acids exert a more pronounced effect on microbial community structure than glucose. This finding is consistent with the observation reported by Wiesenbauer et al. [74] that microorganisms exhibit a metabolic preference for organic acids over simple sugars, with organic acids more readily inducing functional reorganization within microbial communities.

As hypothesized in H2, different exogenous additives recruited distinct functional microbial assemblages. In terms of microbial enrichment, research has revealed that conventional fertilization preferentially enriches microorganisms closely associated with the nitrogen cycle. The primary biomarkers comprise multiple taxonomic units within the phylum Nitrospirota, including the class Nitrospiria, the order Nitrospirales, the family Nitrospiraceae, and the genus *Nitrospira*. Nitrospirota is a phylum of nitrifying bacteria found in soil. Traditionally known for its role in nitrite oxidation, recent research by Hayatsu et al. [75] has revealed that certain Nitrospira species within this phylum are capable of complete ammonia oxidation (Comammox), directly converting ammonium (NH_4_^+^) to nitrate (NO_3_^−^). This finding suggests that NPK treatment has the potential to significantly enhance soil nitrification processes.

The supplementation of glucose and citric acid has been demonstrated to significantly impact the enrichment of multiple beneficial microbial groups [51]. Among these, the Pseudomonadaceae family showed particularly pronounced enrichment; its member, *Pseudomonas chlororaphis*, a species well known for its biocontrol potential [76], was particularly enriched. Concurrently, *Acinetobacter*, which possesses diverse metabolic functions, emerged as a key biomarker in response to this treatment [77]. The enrichment of these biocontrol-related taxa under glucose and citric acid treatments may facilitate the suppression of soil-borne pathogens, thereby promoting root health and supporting the improved plant biomass observed in Section 3.1. We hypothesize that these treatments enhance disease suppression and plant growth by fostering a beneficial microbial microenvironment.

The application of organic fertilizer resulted in a substantial enrichment of multiple members of the Bacteroidetes phylum, including Chitinophagales, Chitinophagaceae, Flavobacteriales, Flavobacteriaceae, and *Flavobacterium*. These taxa are renowned for their efficient capacity to degrade complex organic compounds, including chitin and proteins [78]. Consequently, their enhanced decomposition capacity likely accelerated the mineralization of organic substrates, increasing the availability of nutrients such as nitrogen and phosphorus [79]. This mechanism provides a plausible explanation for the superior fruit weight (6.53 kg) and improved sugar content observed under NPKM treatment. We hypothesize that the NPKM treatment may have strongly stimulated soil carbon cycling processes, enhancing the potential for organic matter decomposition and nutrient release [80].

Moreover, amino acid and acetic acid treatments each enriched their respective characteristic biomarkers. For instance, acetic acid treatment was found to be conducive to the proliferation of Ectobacillus. Under amino acid treatment, two key bacterial families exhibited an enrichment trend: Rhizobiaceae, whose core characteristic is the potential for biological nitrogen fixation [81]; and Comamonadaceae, which is capable of participating in the degradation of various carbon sources as well as denitrification processes [82]. These findings indicate that different exogenous inputs do not merely alter microbial abundance but specifically enrich groups possessing entirely distinct ecological functions.

Consistently, the enrichment of Rhizobiaceae under amino acid treatment indicates enhanced biological nitrogen fixation potential, which likely contributed to the improved nitrogen nutrition and fruit quality parameters observed in the NPKGA treatment (Section 3.2). In summary, this comprehensive analysis indicates that exogenous additives, particularly organic fertilizers and organic acids, can influence the rhizosphere microenvironment of watermelon in continuous cropping systems. This enhancement is achieved through a synergistic physicochemical–microbial mechanism, thereby promoting soil fertility and crop productivity. The findings provide a theoretical basis for the ecological regulation of continuous cropping obstacles in watermelon and offer practical guidance for the rational selection of regional soil conditioners.

## 5. Conclusions

This study systematically elucidates the mechanisms by which different exogenous additives—particularly integrated organic–inorganic amendments centered on organic fertilizer—enhance soil health, promote watermelon growth, and improve fruit quality. Different additives exhibited distinct regulatory patterns on microbial activity: organic fertilizer enriched beneficial bacteria, while organic acids and amino acids stimulated specific soil enzymes. A dynamic coupling was observed between microbial shifts and soil nutrient release, with bacterial and fungal communities correlating with key soil properties (salts, pH, and nitrogen). These microbial changes were linked to improvements in soil organic matter, total nitrogen, and crop performance. Practically, organic fertilizer was associated with higher fruit weight and increased soil organic matter, while organic acids enhanced reducing sugar content, and amino acids improved catalase activity. These findings provide a basis for selecting appropriate additives to mitigate continuous cropping obstacles.

As these findings are from a single-season experiment, several future research directions are warranted: (1) long-term studies integrating metagenomic and multi-omics approaches are needed to further elucidate root–microbe interactions and validate the persistence of the observed effects; (2) the optimal application rates and timing for each exogenous additive should be determined to maximize their agronomic benefits; (3) the combined application of multiple additives (e.g., organic fertilizer + amino acids) warrants investigation to explore potential synergistic effects on crop performance and soil health. Such multi-faceted investigations will contribute to developing precise, sustainable soil management strategies for continuous watermelon cropping systems.

## Figures and Tables

**Table 1:** Description of the eight treatments and experimental design.

Treatment	Description	Application Rate
CK	No fertilizer	-
NPK	Chemical fertilizer only	N 225 kg/ha, P_2_O_5_ 120 kg/ha, K_2_O 90 kg/ha
NPKM	NPK + sheep manure	Manure: 18,525 kg/ha
NPKC	NPK + glucose	Glucose: 150 kg/ha
NPKOA	NPK + oxalic acid	Oxalic acid: 150 kg/ha
NPKGA	NPK + amino acids	Amino acids: 150 kg/ha
NPKCA	NPK + citric acid	Citric acid: 150 kg/ha
NPKAA	NPK + acetic acid	Acetic acid: 150 kg/ha

The sheep manure used in NPKM was composted for approximately 3 months before application. Based on typical values reported in the literature, the manure contained approximately 24–27% organic matter, 0.5–0.8% N, 0.4–0.6% P_2_O_5_, and 0.4–1.0% K_2_O [17,18,19,20].

**Table 2:** Analysis of Watermelon Fruit Quality.

Sample	CK	NPK	NPKM	NPKC	NPKOA	NPKGA	NPKCA	NPKAA
Moisture content (%)	89.17 ± 0.47 ^a^	88.77 ± 0.34 ^a^	88.53 ± 0.03 ^a^	89.17 ± 0.61 ^a^	88.27 ± 0.37 ^a^	88.57 ± 0.30 ^a^	88.17 ± 0.26 ^a^	88.93 ± 0.24 ^a^
Dry matter (%)	10.83 ± 0.47 ^a^	11.23 ± 0.34 ^a^	11.47 ± 0.03 ^a^	10.83 ± 0.61 ^a^	11.73 ± 0.37 ^a^	11.43 ± 0.30 ^a^	11.83 ± 0.26 ^a^	11.07 ± 0.24 ^a^
Soluble solids (%)	10.20 ± 0.50 ^a^	10.60 ± 0.35 ^a^	10.53 ± 0.79 ^a^	10.43 ± 0.64 ^a^	11.27 ± 0.69 ^a^	10.83 ± 0.19 ^a^	10.97 ± 0.12 ^a^	10.77 ± 0.38 ^a^
Vitamin C (mg/100 g)	1.26 ± 0.16 ^a^	1.13 ± 0.11 ^a^	1.29 ± 0.04 ^a^	1.14 ± 0.28 ^a^	0.95 ± 0.33 ^a^	1.58 ± 0.18 ^a^	1.52 ± 0.33 ^a^	1.25 ± 0.06 ^a^
Total acidity (g/kg)	1.23 ± 0.23 ^a^	1.92 ± 0.30 ^a^	2.08 ± 0.45 ^a^	1.78 ± 0.21 ^a^	2.03 ± 0.31 ^a^	2.02 ± 0.07 ^a^	1.81 ± 0.20 ^a^	1.87 ± 0.19 ^a^
Potassium content (mg/kg)	2189.33 ± 343.38 ^a^	2300.33 ± 92.35 ^a^	2793.33 ± 131.38 ^a^	2865.33 ± 146.03 ^a^	2679.33 ± 196.00 ^a^	2315.00 ± 192.67 ^a^	2329.33 ± 201.84 ^a^	2683.00 ± 301.89 ^a^
Protein (g/100 g)	0.96 ± 0.05 ^a^	1.00 ± 0.03 ^a^	1.02 ± 0.05 ^a^	1.03 ± 0.13 ^a^	1.04 ± 0.07 ^a^	1.06 ± 0.05 ^a^	0.99 ± 0.04 ^a^	1.07 ± 0.06 ^a^
Phosphorus content (mg/kg)	286.00 ± 58.80 ^a^	373.33 ± 22.58 ^a^	418.33 ± 64.85 ^a^	367.00 ± 37.32 ^a^	349.33 ± 24.39 ^a^	325.67 ± 35.79 ^a^	340.67 ± 52.78 ^a^	358.00 ± 50.85 ^a^
Reducing sugar (g/100 g)	7.30 ± 0.65 ^a^	8.10 ± 0.35 ^a^	7.97 ± 0.78 ^a^	7.93 ± 0.70 ^a^	9.23 ± 1.08 **^a^**	8.13 ± 0.49 ^a^	8.13 ± 0.44 ^a^	9.03 ± 0.73 ^a^
Fructose (g/100 g)	6.00 ± 1.03 ^a^	3.13 ± 0.33 ^b^	7.07 ± 0.84 ^a^	6.17 ± 0.35 ^a^	4.63 ± 0.27 ^ab^	4.93 ± 0.59 ^ab^	6.47 ± 0.68 ^a^	6.77 ± 1.43 ^a^
Glucose (g/100 g)	3.47 ± 0.94 ^a^	1.27 ± 0.07 ^b^	2.70 ± 0.68 ^ab^	3.00 ± 0.47 ^ab^	1.27 ± 0.27 ^b^	2.23 ± 0.13 ^ab^	2.57 ± 0.27 ^ab^	2.90 ± 0.70 ^ab^
Sucrose (g/100 g)	0.93 ± 0.32 ^c^	1.93 ± 0.03 ^bc^	4.00 ± 0.85 ^a^	2.47 ± 0.52 ^abc^	2.53 ± 0.59 ^abc^	2.83 ± 0.50 ^bc^	3.57 ± 0.41 ^bc^	3.50 ± 0.60 ^bc^

Values represent the means ± standard errors (*n* = 3). Values with the same lowercase letter are not significantly different (*p* < 0.05). Data within a column followed by different lowercase letters indicate a significant difference at the 0.05 probability level according to protected LSD tests.

## Data Availability

The original contributions presented in this study are included in the article. Further inquiries can be directed to the corresponding author.
